# Construction of Direction Selectivity through Local Energy Computations in Primary Visual Cortex

**DOI:** 10.1371/journal.pone.0058666

**Published:** 2013-03-15

**Authors:** Timm Lochmann, Timothy J. Blanche, Daniel A. Butts

**Affiliations:** 1 Department of Biology and Program in Neuroscience and Cognitive Science, University of Maryland, College Park, Maryland, United States of America; 2 Redwood Center for Theoretical Neuroscience, University of California, Berkeley, California, United States of America; 3 Helen Wills Neuroscience Institute, University of California, Berkeley, California, United States of America; University of Southern California, United States of America

## Abstract

Despite detailed knowledge about the anatomy and physiology of neurons in primary visual cortex (V1), the large numbers of inputs onto a given V1 neuron make it difficult to relate them to the neuron’s functional properties. For example, models of direction selectivity (DS), such as the *Energy Model,* can successfully describe the computation of phase-invariant DS at a conceptual level, while leaving it unclear how such computations are implemented by cortical circuits. Here, we use statistical modeling to derive a description of DS computation for both simple and complex cells, based on physiologically plausible operations on their inputs. We present a new method that infers the selectivity of a neuron’s inputs using extracellular recordings in macaque in the context of random bar stimuli and natural movies in cat. Our results suggest that DS is initially constructed in V1 simple cells through summation and thresholding of non-DS inputs with appropriate spatiotemporal relationships. However, this *de novo* construction of DS is rare, and a majority of DS simple cells, and all complex cells, appear to receive both excitatory and suppressive inputs that are already DS. For complex cells, these numerous DS inputs typically span a fraction of their overall receptive fields and have similar spatiotemporal tuning but different phase and spatial positions, suggesting an elaboration to the Energy Model that incorporates spatially localized computation. Furthermore, we demonstrate how these computations might be constructed from biologically realizable components, and describe a statistical model consistent with the feed-forward framework suggested by Hubel and Wiesel.

## Introduction

The primary visual cortex (V1) takes an important position in visual processing, receiving both feed-forward input from the lateral geniculate nucleus (LGN) and cortico-cortical connections, and forms the basis for further processing in higher cortical areas [1]. The diversity of connections to a given V1 neuron confers selectivity to a rich repertoire of stimulus features, such as orientated bars and direction of motion [2], [3].

Direction selectivity (DS) is often found in cells whose response is invariant to the spatial phase of a moving grating, and thus is a relatively accessible example that demonstrates more general principles of hierarchical computation. Namely, selectivity to a higher-order visual feature (motion direction) is paired with invariance to other aspects of the visual stimulus irrelevant to DS [4]: in this case spatial phase. Furthermore, such a representation in V1 forms the basis of further processing along the dorsal stream [5], which becomes increasingly invariant to all but motion [6]. The change in response properties from phase-dependent simple cells to phase-invariant complex cells within V1 therefore has been the subject of a range of modeling studies, beginning with Hubel and Wiesel’s feed-forward hypothesis [7], which suggested that phase-invariant complex cells pool over a distribution of similarly tuned (but phase-dependent) simple cells. This original hypothesis has been reflected in the “Energy Model” [8], [9], whereby DS phase-invariance is constructed by a *quadrature pair* of simple cell filters: adding the squared output of two simple cell receptive fields that thereby reflects the phase-invariant power (“motion energy”) at a particular spatiotemporal frequency.

Beyond an abstract classification of both inputs and corresponding computation [10]–[13] it has, however, been very difficult to validate such models experimentally, in part due to the large number of inputs onto a given V1 neuron. Here we use a statistical approach based on extracellularly recorded V1 spike trains, which can infer both the properties of individual inputs and describe how they are combined to yield the properties of the recorded neuron. This approach is based on spike-triggered covariance analysis (STC, [14], [15]), combined with maximum-likelihood methods [16], [17], and advances previous work in two ways. First, we extend STC to find a set of “localized” features that the neuron is selective to. Unlike many of the STC filters, which are multi-lobed and spatially extensive, the resulting localized filters are more consistent with those expected of classical simple cells [9], [18], and are self-consistent with those observed across the broad dataset that we analyze. Second, applying this method to simple and complex cell responses allows for the inference of how different types of DS are constructed: while the responses of a few simple cells are computed through summation and thresholding of non-DS “LGN-like” inputs, the majority of DS simple cells already appear to receive DS input. Similarly, complex cells are found to receive a relatively balanced pool of inputs that results in the observed phase invariance typical of complex cells. Based on this new method to reliably infer characteristic input properties and associated nonlinearities, our results both reconcile previous observations of V1 processing and suggest an implementation of the Energy Model for V1 DS based on pooling over local stimulus energy through a biologically plausible computation.

## Materials and Methods

### Experimental Recordings with Random Bar Stimuli

This work is primarily based on a dataset recorded by N. Rust, with full details described in [15]. Briefly, following surgery, male macaque monkeys were anesthetized with 4–12 µg/kg/hr sufentanyl citrate and paralyzed with Vecuronium bromide (Norcuron, Organon), administered intravenously at 0.15 mg/kg/hr. Single units with receptive fields centered between 3° and 20° from the fovea were recorded in V1 using quartz-glass electrodes while visual stimuli were presented on a CRT monitor. All experiments were performed in compliance with the National Institutes of Health Guide for the Care and Use of Laboratory Animals and within the guidelines of the New York University Animal Welfare Committee.

After determination of a cells preferred direction, spatial and temporal frequency, black and white bars matched to these preferences were presented at 100 Hz and a mean luminance of 33 cd/m^2^ at spatial locations determined pseudo-randomly by a binary *m*-sequence [19]. For a subset of cells, the Modulation Index (MI = F1/DC, i.e. the first Harmonic F1 divided by the average response level) and the Direction Selectivity Index (DSI) were measured using drifting gratings. The DSI is defined as [Rp-Rnp]/[Rp+Rnp] with Rp and Rnp being the average firing rates at the preferred and non-preferred directions [20]. Depending on responsiveness of the cell, recordings lasted 15–80 min and contained 3,000–250,000 spikes.

### Experimental Recordings with Naturalistic Stimuli

In order to confirm our results in a more complex stimulus context, we also used data from anesthetized, paralyzed cats, described in detail in [21]. Extracellular multi-neuron spike trains were recorded with a 54-channel high-density silicon polytrode array implanted in the primary visual cortex of anesthetized (Isofluorane, 0.25–1.5%) and paralyzed (Pancuronium bromide (2 mg/kg) adult cats. All experiments were performed in accordance with the guidelines established by the Canadian Council for Animal Care.

Visual stimuli were presented on a CRT monitor at a resolution of 800×600 pixels and a refresh rate of 200 Hz. Neural responses were evoked with drifting sinusoidal gratings (for computing MI and DSI), 1/*f* ‘pink’ spatiotemporal noise, and dynamic natural scene movies that were captured by attaching a small CCD camera to a cat's head while it roamed freely in the woods [22]. All stimuli were presented at 50 Hz, subtended 12 degrees of visual angle, and had a mean luminance of 52 cd/m^2^. The models in this study were applied to spike trains recorded during the pink noise and natural movie stimulation, a combined duration of 26 minutes. Neurons had receptive fields within 10° of the area centralis, and fired between 2,300∼42,000 spikes.

### Spike-triggered Analyses

**Figure 1:**
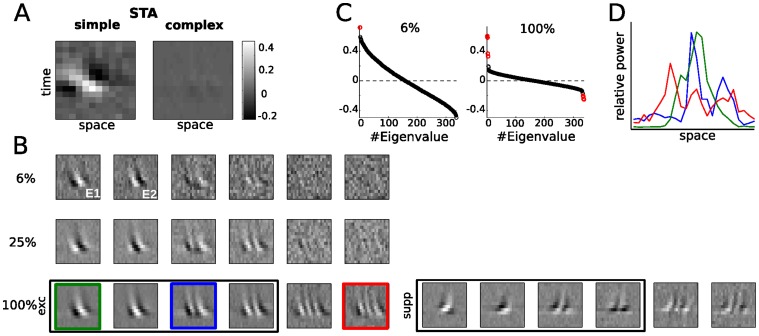
Spike triggered analysis of direction selective simple and complex V1 cells. **A.** The Spike Triggered Average (STA) for a simple cell (*left*) and a complex cell (*right*), shown as “*x*-*t*” plots: the *horizontal axis* corresponds to the spatial location perpendicular to a bar oriented in the neuron’s preferred direction, and the *vertical axis* indicates the time preceding a spike (10–140 ms). The grayscale value represents the magnitude of the STRF, indicating how strongly an increased/decreased luminance value at a spatiotemporal location increases or decreases the probability to spike. The tilt in the simple cell STA (*left*) demonstrates that it is direction selective, but the STA cannot capture the direction selectivity of the complex cell (*right*). **B.** V1 DS complex cells are sensitive to large numbers of motion features but the specific number of significant STC filters strongly depends on the amount of available data. With increasing data, more and more filters are detected (lines correspond to 8423, 28435, and 117097 spikes, respectively). However, the filters added (both “excitatory” and “suppressive”) with more data do not resemble those first detected, having higher spatial frequencies and less spatial localization (suppressive filters for smaller amounts of data are present but not shown). Note that the suppressive filters (*bottom row, right*) have opposite direction selectivity from the excitatory filters (left). The thin frame indicates the filters with the largest positive and negative eigenvalues, corresponding to the *red dots* in (C). **C.** The corresponding eigenvalue spectra of the spike-triggered covariance (STC) matrices estimated from 6% and 100% of the data indicate two vs. eight significant filters (*red circles*), respectively. **D.** The profiles of temporal power along each pixel position for the three STRFs indicated in **B** (last row) are diverse, ranging from relatively localized and unimodal to spatially extensive and multimodal.

To characterize the relationship between the stimulus and a given V1 neuron’s response to the random-bar stimulus, we began by computing the spike-triggered average (STA), which gives the linear (first order) model of this relationship. The STA was computed as the average 14 stimulus frames preceding a spike across all spatial positions, and is displayed as a two-dimensional *x-t* plot (Fig. 1A). Because most V1 neurons appear to respond to more than a single stimulus feature [15], the STA alone cannot fully represent their stimulus selectivity, and thus we used spike-triggered covariance (STC) analysis [14], [15], [23], [24] which is sensitive to information about spatiotemporal stimulus correlations, i.e., second-order effects. The stimulus covariance matrix was computed over the entire experiment, and this overall covariance was subtracted from the covariance of stimuli triggering spikes. The eigenvectors of this matrix provide a concise abstract description of the cell’s selectivity and are naturally ordered by their associated eigenvalues. They represent the “directions” in the stimulus space (which we refer to as “STC filters”) that can be directly related to the probability that the cell will fire. Note that we did not enforce the STC filters to be orthogonal to the STA, which is a choice made in some applications [24] but not others [23]. In our application, enforcing orthogonality does not affect our results, particularly considering the often-negligible STA of complex cells. The resulting STC filters that have eigenvalues significantly different than zero are classified as “excitatory” (increased variance) or “suppressive” (decreased variance) [24].

Which of these eigenvectors effectively contribute to a cell’s selectivity can be decided by testing whether they significantly improve a model’s ability to predict a new data set [24], [25] or via computationally less demanding heuristics based on the distribution of eigenvalues [26]. Because our results do not strongly depend on the exact number of included STC dimensions, we took inclusive sets of STC filters consisting of the eigenvectors with the *N*_pos_ largest and *N*_neg_ smallest eigenvalues, with *N*_pos_ = 2–8 for simple cells, 4–10 for complex cells, *N*_neg_ = 0–10 for simple cells, 4–10 for complex cells.

### Dimensionality Reduction and Spatiotemporal Whitening of Natural Movies

For the correlated noise sequences and naturalistic movies, it was necessary to first “whiten” the stimulus [27] in order to eliminate correlations in the stimulus, and reduce the dimensionality of the original stimulus space (*d*_space_*d_time_ = *d*_st_ 1024*6 = 6144). To this end, the stimuli **s** *_t_* were “temporally embedded” into a design matrix **S**, such that each row of **S** contained the preceding spatiotemporal stimulus that might contribute to the response at time *t*, including the 6 preceding time bins across all spatial positions. Principal Component Analysis (PCA) was then used to generate the “whitened” design matrix, to which spike-triggered analyses could be applied [27], [28]. More specifically, the whitened matrix **Z** is obtained using **Z** = **SV**'**D** ^−1/2^ where **V** is an *N*_st_x*M* matrix containing the first *M* eigenvectors of the stimulus covariance matrix **S'S**, and **D** is a diagonal matrix containing the corresponding eigenvalues. Such a reduced representation **Z** requires less data to obtain reliable estimates of the STC filters {**b** *_i_*} and M = 600 is chosen to balance reliability of estimated filters against resolution of the resulting filters. With this approach, the STC filters can be computed in the whitened space {**b** *_i_*_,w_} [28]. Because of the noise inherent in the large numbers of dimensions in this analysis, we found that performance improved by accentuating stimulus dimensions with large variability, and used **b** *_i_* = **VD** ^1/2^ **b** *_i_*_,w_, effectively smoothing the resulting estimates.

### Determination of Localized Filters from STC Filters

The STC filters **b** *_i_* provide a succinct basis to describe a neuron’s selectivity to different inputs. However, although they provide an efficient representation of the stimulus subspace related to the neuron’s response, the spatiotemporal structure of these basis vectors suggests that they are not describing synaptic inputs, raising the question as to whether there is an alternative description of this stimulus subspace using a set of filters {**k_β_**} that more closely resemble the types of inputs cortical neurons are known to receive. Such filters would be contained in the same stimulus subspace and therefore be representable by linear combinations **k**_β_ = Σ_i_ β_i_ **b**_i_ of the *M* STC basis vectors B[**b**_1_, °K, **b**_M_]. In addition to existing within the STC subspace, here we also incorporate prior assumptions about the spatiotemporal profiles of the filters [29], thereby providing additional information about the putative inputs to the cell. To this end, we devised a method that finds spatially localized excitatory and suppressive filters consistent with the observed STC results. The method can be applied separately to the subspaces spanned by just excitatory or just suppressive filters, or to the combined space. Because we expect that excitatory and suppressive effects stem from different cell types, we applied this method to each space separately, which is easier and slightly more robust in general, while yielding qualitatively similar results.

To identify localized filters in the space spanned by STC filters, we find the linear combinations of the STC filters that have the most compact spatial profile centered on each spatial location *c*. To characterize the spatial profile of a particular filter in the STC subspace, we define the *temporal power* of the filter ***k_β_*** at position *x* to be *R* ***_β_*** (*x*)* = 1/T* Σ*t*[<***k_β_***(*x,t*)>*_t_ –* ***k_β_***(*x,t*)]*^2^.* Starting from randomly initialized **β** we then numerically minimize the cost function *L*(*c*,**β**) = Σ*_x_R* **_β_**(*x*)*(*c*-*x*)^2^ with respect to **β** under the constraint Σ_i_ **β**_i_^2^ = 1. The cost function determines the degree to which **k_β_** is localized around the spatial location *c,* because it minimizes the distance (*c*-*x*) to the target location weighted by the power profile *R* **_β_**(*x*). Beyond this term quantifying the spatial localization of temporal power, no other constraints were imposed when optimizing this cost function. To avoid local minima, we took the most localized filter from 20 different initializations of **β** for each of 40 spatial positions *c* evenly covering the stimulus domain. We find that our results are not sensitive to the exact cost function used, and other measures of localization give very similar results. Note that this method also can extract multiple localized filters at a specific location, by projecting out the first filter and then searching for the next most localized filter at the same location in the resulting orthogonal subspace.

### Nonlinear Modeling

Firing responses of cells in sensory areas resulting from visual stimulation can be predicted by models that cascade stages of filtering and nonlinear combination [30], [31]. To predict individual spike trains of cells in V1, we used a Generalized Nonlinear Model (GNM, [17]) based upon the filters derived from the STC and localization procedures described above. For such a model, the time varying firing rate is given as *r*(*t*) = *F*[Σ_i_Σ_τ_(**h**_iτ_*f_i_(**k**_i_*s_t-τ_))-θ], where *F*[·] = log[1+exp(·)] is the spiking nonlinearity that maps the summed output of the individual terms to a firing rate. The terms *f*_i_(**k**_i_*s_t_) are nonlinear functions *f*_i_ of the stimulus **s**_t_ convolved with the different filters **k**_i_, and the parameters **h**_iτ_ allow for modeling of temporal integration of these nonlinearly filtered inputs *f*_i_(**k**_i_*s_t-τ_). This model class represents an extension of Generalized Linear Models (GLM, [31]), which is similar to Generalized Additive Models [32], and given the filter estimates **k**_i_, allows for simultaneous estimation of their nonlinear impact on the firing rate via optimization of the model likelihood [16], [17]. The GNM framework easily incorporates prior assumptions about the estimated parameters and we included penalty terms enforcing smoothness of temporal integration and nonlinear combination [17].

### Selection of Relevant Localized Directions Using Regression with a Sparseness Prior

The above-mentioned method for the identification of localized filters generates a large number of candidate filters. The subset of features most effectively describing the cells’ selectivity can then be determined using a regularization approach. We filtered the stimulus with each of the localized filters, along with its negative counterpart, and used logistic regression with a sparseness prior [33], [34] on their rectified output. The optimal value for the sparseness parameter was determined via cross-validation, typically resulting in a slightly larger set of localized filters than corresponding STC filters. The nonlinearities for the resulting set of filters were subsequently fit using the GNM framework.

### Prediction of the Direction Selectivity Index (DSI)

The spatiotemporal selectivity of the STC and localized filters described above can also be used to predict classical measures of a neuron’s selectivity such as its Direction Selectivity Index (DSI) and Modulation Index (MI). As described in [35], the DSI of simple cells can be predicted from spatiotemporal receptive fields such as the STA, STC filters, or localized filters as: DSI_RF_ = (Q1–Q2)/(Q1+Q2), where Q1 and Q2 are the upper right and left quadrants of the two dimensional Fourier transformation of the receptive field.

### Construction of Direction Selective Features from Spatiotemporally Separable Inputs

The STC filters for DS simple and complex cells exhibit oriented *x*-*t* plots that are spatiotemporally inseparable (i.e., cannot be constructed via simple multiplication of a spatial and a temporal kernel, see Figs. 1A,B). As described in [8], such DS filters can, however, be constructed by addition and subtraction of spatiotemporally separable inputs. We use singular value decomposition (SVD, [34]) to decompose such DS filters into putative separable filters, yielding non-DS inputs that could explain the observed DS filter. Furthermore, while phase-invariant DS responses of complex cells cannot be described by just one simple DS filter, they can easily be constructed via nonlinear combination of two matched DS filters [8].

## Results

Direction selectivity of V1 neurons is classically characterized using simple motion stimuli such as drifting gratings or bars, where the firing rate in response to different motion directions can be unambiguously measured. Such stimuli have relatively simple spatiotemporal properties and therefore would not be able to constrain models with many parameters, such as those describing the construction of DS from component spatiotemporal elements. The use of more complex stimuli can provide more information to fit models with a larger number of parameters, but of course requires more advanced statistical approaches to reliably estimate them. Using such approaches, we will first analyze V1 spike trains recorded in the context of “random bar” stimuli, comprised of random combinations of black and white bars covering the classical receptive field at the neuron’s preferred orientation [15]. While the random bar stimulus does not confine the analysis of DS to the restricted subspace of moving gratings, it is still much simpler than the natural stimuli we consider later, and therefore allows for the derivation of a detailed picture of V1 neurons’ sensitivity to spatiotemporal patterns in a simplified stimulus context.

For some V1 neurons, the linear receptive field - calculated in this context from the spike-triggered average (STA) - demonstrates the neuron’s selectivity for a particular direction. For the simple cell in Figure 1*A* (*left*), the STA shows that the neuron’s preferred spatial position systematically changes with latency, resulting in a tilted spatiotemporal profile. Likewise, the neuron's spatial and temporal frequency preferences can also be derived from this *x-t plot* [35] using the horizontal and vertical widths of the STA features. In contrast, the complex cell’s STA shows no structure and fails to describe its robust response to motion direction (Fig. 1*A*, *right panel*). The lack of structure in the STA is emblematic of the *phase-invariance* of complex cells, and results from the fact that the neuron responds equally well to black or white bars moving in the preferred direction, which - because they both evoke spikes - average to a uniformly gray STA. Thus, nonlinear operations are necessary in order to respond to both a stimulus and its opposite, and DS complex cells therefore require nonlinear analyses to understand the origins of their selectivity [14], [15].

### Nonlinear Analysis of Direction Selectivity in Simple and Complex Cells

While the first-order (STA) characterization of stimuli driving complex cell spikes fails due to the overlapping, roughly equal response to light and dark stimuli, complex cell selectivity can be detected using higher-order statistical analyses, such spike-triggered covariance (STC) analysis. This approach can detect selectivity to stimulus features based on the second-order relationship between stimulus and spikes. For example, responding to a spatiotemporal pattern and its opposite equally will result in a zero spike-triggered average stimulus, but is detectable by looking at the direction where the variance of the spike-triggered stimulus is highest. The first V1 study to apply STC analysis to random-bar data found two spatiotemporal filters underlying complex cell responses [14], which is consistent with our measurements when using only 6% of data (8,423 spikes) (Fig. 1*B*, top row, 1*C* left). In this case, STC analysis finds that complex cell selectivity is well described by a *quadrature pair* of filters (Fig. 1*B*, filters E1 and E2), representing two simple-cell-like DS filters with roughly the same spatiotemporal tuning, but 90 degrees phase difference. They both have a clear spatiotemporal tilt, resulting in selectivity for direction of motion. Such selectivity for complex cells was explicitly predicted by the Energy Model [8], and directly measured with this approach in [14].

Unfortunately, this easily interpretable picture of complex cell selectivity in the context of the Energy Model becomes less clear with more data (Fig 1*B*). In fact, the much richer description of complex cell selectivity demonstrated in [15] appears to be simply related to the amount of data - applying STC analysis to the full recording (27 minutes, 117,097 spikes) reveals many more spatiotemporal filters contributing to the neuron’s response (Fig. 1*B* bottom row and 1*C* right panel).

The much longer recordings in [15] in fact reveal many more spatiotemporal elements in a majority of recorded neurons: nearly all complex cells have more than two filters, with more than half having four or more excitatory filters and up to nine suppressive filters [15]. These additional filters typically exhibit higher spatial frequency and broader spatial extent than the filters found with less data.

Is the Energy Model then just an approximation to a more ornate computation performed by the neuron using many multi-lobed filters with a range of spatiotemporal frequencies? Furthermore, what is the source of these spatially extensive, multi-lobed spatiotemporal elements? Such spatiotemporal filters with large numbers of subfields (Fig. 1*B, D*) are not seen for simple cells in our dataset (see below), and in general not typical for V1 simple cells (*[36]*, *[37]*; for quantitative analysis of RF properties see [18]). However, here we will show that the computation captured by STC analysis is in fact consistent with a more physiologically realistic elaborated version of the Energy Model, as well as the diversity of spatiotemporal filters observed in this dataset [15], and arises from the spatial scale of the inputs to a complex cell, compared with the extent of the overall receptive field.

### Localized Filters Yield a Consistent Model for the Construction of Direction Selectivity

**Figure 2:**
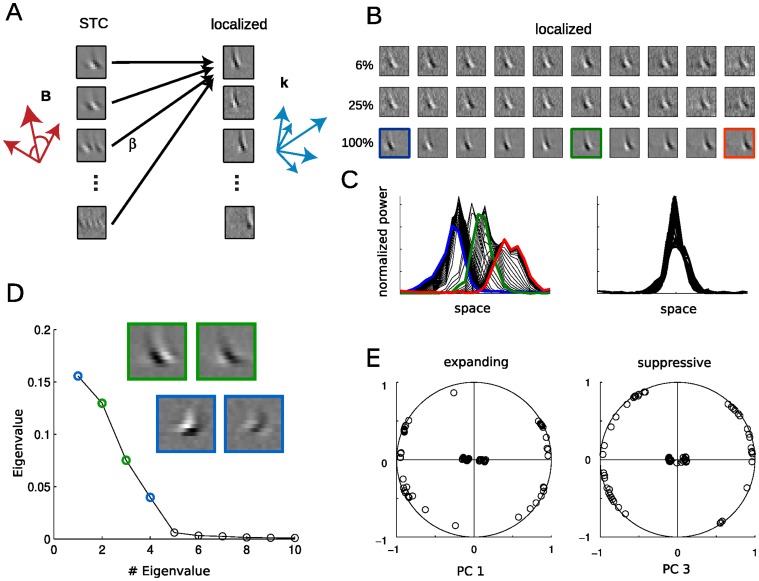
Localized filters provide a biologically plausible alternative description of the STC subspace. **A.** The subspace defined by the STC filters can be used to find a set of localized filters, where each localized filter (*right*) is a linear combination of the set of STC filters (*left*). The small filters (STC and localized) are taken from 1B and 2B (last row) and exemplify the different features that can be used to represent a cells sensitivity. *Red arrows* illustrate an orthogonal vector space defined by the STC filters, *while blue arrows* illustrate a larger number of non-orthogonal localized filters that span the same subspace. **B.** Localization detects a large number of filters within the STC subspace, and 10 (from 47) localized solutions spanning the RF are shown. As more data is used for the analysis, the properties of the filters remain relatively constant, but their features become less polluted by noise. The localized solutions show similar spatiotemporal sensitivity, with the main differences between the filters amounting to differences in position and phase. **C.** The power profiles of 47 localized excitatory filters (*left*) have been aligned (*right*) to compare the similarity of their spatial envelopes. **D.** The 47 excitatory and 47 suppressive localized solutions form 2 homogeneous populations of spatially confined features that are succinctly described by four eigenvectors - two spanning the excitatory subspace, and two spanning the suppressive subspace - as determined by PCA applied to the set of spatially shifted localized filters in D. **E.** The filters are well represented by these PCA eigenvectors (98% exp, 97% suppressive) and evenly cover the range of possible combinations of these vectors.

Understanding the computation suggested by the STC analysis requires considering the STC filters as a coordinate system for the *stimulus subspace* that the neuron is sensitive to, rather than considering the filters individually [15], [25], [38]. Just as one can rotate a coordinate system, one can describe this stimulus subspace with a different set of filters in this subspace (*e.g.*, Fig 2*A*), raising the possibility that there is an alternative description of the neuron’s sensitivity that is consistent with the STC analysis, but maps to a physiologically more plausible description of the neuron’s inputs.

We search the subspace determined by STC for filters that more closely resemble those seen for simple cells in the dataset (see below), using a cost function that favors filters that are as spatially localized as possible at any given location (see Methods). The excitatory and suppressive subspaces are analyzed separately, resulting in corresponding sets of excitatory and suppressive localized filters (for examples of excitatory filters, see Fig. 2*B* last row). These filters provide an alternative and more localized representation of the excitatory and suppressive subspaces identified by STC. In contrast to the STC solution, this set of filters is not constrained to be orthogonal, and thus there can be many more localized filters than STC filters.

Although the localization penalty could in principle lead to wildly different types of filters found at each location, the set of localized filters centered at each location is typically composed of filters with very similar spatiotemporal structure (Fig. 2*B*), including nearly identical spatial frequency and speed selectivity. Furthermore, their power profiles have similar shapes (Fig. 2*C*), and tile the larger area of the neuron’s receptive field. The similarity of the localized filters across space is further demonstrated by performing principal component analysis (PCA) on the spatially aligned localized features, which reveals that the entire set of excitatory or suppressive filters can each be well represented by two basis vectors (Fig. 2*D,E*). These basis vectors have nearly identical spatiotemporal profiles yet are orthogonal to one another, constituting a quadrature pair. This suggests that motion energy is computed at a smaller spatial scale and pooled across the neuron’s receptive field, and that the additional STC filters revealed by more data reflect the multiple spatial locations that such pooling occurs over.

In addition to providing a concise description of V1 complex cell computation as a “Localized Energy Model”, this description is more robust to the amount of data used for analysis. As more data is used to produce the STC filters, new spatiotemporal selectivity emerges in the additional resulting filters, which have more lobes and less spatial localization (compare Fig. 1*B* and *D*). In contrast, the localized filters tend to maintain similar spatiotemporal properties, while their features become less noisy (Fig. 2*B*). The stability of the properties of the localized filters as more data is added suggests they are capturing the actual integrative properties of the neuron, which would not be expected to change with the length of the experiment.

### Nonlinear Construction of Direction Selectivity

The construction of complex cell computation depends both on the spatiotemporal elements that are input, and the nonlinear computations performed on these inputs. In fact, a crucial component of the Energy Model is the squaring computation applied to its quadrature pair elements, in addition to the spatiotemporal relationships between these elements. Alternatively such computation can be comparable to multiplicative combinations of non-DS cells [8], [39], as in the Reichardt model [40]. With the identification of plausible spatiotemporal elements that comprise complex cell computation, we can now infer how these elements are nonlinearly combined to produce complex cell output, using a previously established nonlinear modeling framework [17].

**Figure 3:**
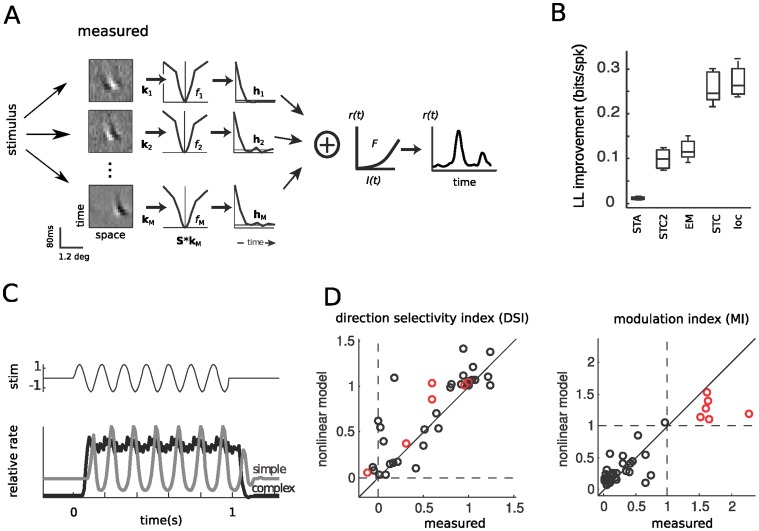
Nonlinear modeling predicts spike timing direction selectivity and modulation index for simple and complex cells. **A.** Schematic of the nonlinear model structure. The stimulus is filtered by a set of receptive fields k*_i_* and further processed by associated nonlinearities *f_i_*(.) and temporal combination term h*_i_*_._ Outputs of multiple expanding and suppressive modules are summed together and converted into a firing rate prediction *r*(*t*) via the spiking nonlinearity *F*. **B.** Cross-validated log-likelihood (*LL*) improvement compared to a model based on the average firing rate. The different levels of STC-based models demonstrate that models with two filters (STC2 and EM) offer large improvements in *LL* over a model based on the STA alone. However, including the full set of detected filters (STC and *loc*) more than doubles model performance. STC2 refers to the model containing only the first two STC filters shown in **1B,** (last row), EM to the two STC filters found for small amounts of data (Fig. **1B**, first row), STC to all filters shown in Fig. **1B** (last row), and *loc* to the localized features (Fig **2B** **,** last row). **C.** Simulated responses of a simple and a complex cell (model as shown in Figure **3A**) to optimally oriented drifting gratings demonstrate the ability of models fit to random bar stimuli to predict response properties to gratings consistent with their cell classification. The responses of the simple cell (*gray*) and complex cell (*black*) are shown relative to the temporal stimulus modulation at a given spatial location (*top*). **D**. Modulation index (MI) and direction selectivity measurements for 51 V1 cells stimulated with optimally oriented drifting gratings (Rust et. al. 2005). The models of all neurons in the study were used to generate predicted Direction Selectivity Index (DSI) and MI (*vertical axis*), which are in agreement with those values measured experimentally (*horizontal axis*), for simple (*red,* correlation coefficient CC = 0.93) and complex (*black,* CC = 0.84) cells, with CC = 0.85 overall. MI is predicted with similar fidelity (*right*, CC = 0.9).

Using this framework, we measure how the output of the relevant filters contribute to the response by fitting nonlinear functions to the output of each filter in order to maximize the likelihood of the model given the observed data (see Methods, Fig. 3*A*). Similar to [33] we use a sparseness prior to first determine this relevant subset from the larger number of localized filters found by the method shown in Figure 2. As these localized filters span the same space as STC, both alternatives allow for the prediction of the spiking response equally well (Fig. 3*B*). Note, however, that in all but the simplest simple cells, we find that models including multiple filters best accounts for the detailed feature sensitivity needed to predict the spiking response. For the example cell shown in Figure 2, taking filters beyond E1 and E2 (Fig. 2B, top row) into account results in a much better prediction of responses to new stimuli than the simple Energy Model (Fig. 3B, compare columns EM vs. STC).

The models comprised of multiple identified filters and their associated nonlinearities can also be used to generate predicted responses to arbitrary stimuli, and therefore to validate their degree of DS and phase invariance (MI) based on the observed responses to moving gratings (Fig. 3*C*). To this end, we fit nonlinear models with the random bar stimulus to then predict individual cell responses to drifting gratings. The predictions are in good agreement with the empirical DSI and MI measurements (Fig. 3*D*; correlation coefficients (CC) between predicted and measured DSI are CC = 0.93 for simple and CC = 0.84 for complex cells, CC = 0.9 across cells for MI).

### The Feed-forward Construction of Direction Selectivity

**Figure 4:**
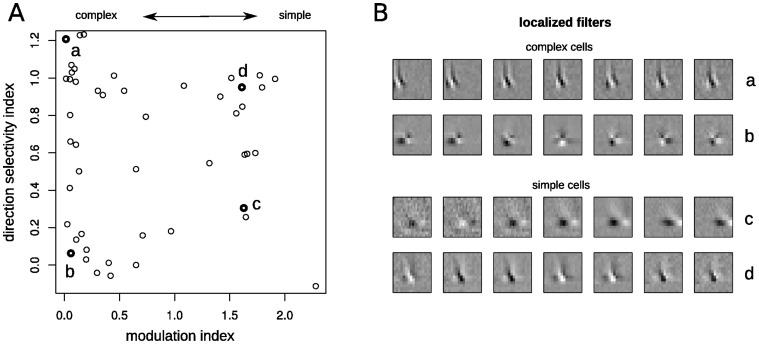
Diversity of response properties across a population of simple and complex cells. **A.** Empirically measured modulation index and direction selectivity values for 51 V1 cells (see Fig. 3, Rust et. al. 2005). The population contains simple and complex cells with varying degrees of direction selectivity. **B.** Both simple and complex cells display sensitivity consistent with multiple localized inputs (shown are 7 localized filters spanning the region RF of the individual cells indicated in **A**).

With the ability to characterize two crucial elements of the construction of DS, the spatiotemporal filters and the nonlinear computation, we can now infer how DS is constructed across the population of V1 neurons in this study, ranging from the “simplest” simple cells to the most complex cells. This range is shown using a subset of 51 cells for which the Direction Selectivity Index (DSI) and Modulation Index (MI) were separately measured using drifting gratings (see Methods) (Fig. 4*A*).

Across the different types of responses - ranging from simple to complex and differing regarding their direction selectivity (Fig. 4*A*), we find STRFs that are consistently explained by pooling of simple localized features (Fig. 4*B*).

Along the continuum of simple to complex cells, these nonlinear characterizations suggest that the contrast-invariant DS of complex cells is built up over one or more intermediate stages and we identify three distinct stages of direction selectivity:

### 1. Direction Selectivity in the ‘Simplest’ Simple Cells can be Constructed from non-DS Inputs

**Figure 5:**
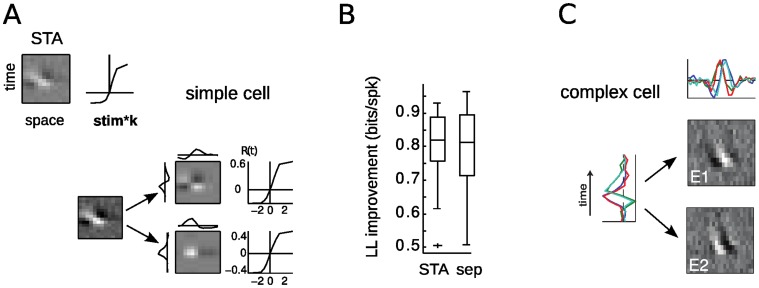
Construction principles of direction selectivity in prototypical simple and complex cells. **A.** An example of a DS simple cell described by a single excitatory filter (*left*). A single nonlinearity associated with this filter, comprising an LN model, offers a good description of its observed response. lower *right:* An alternative model for the simple cell, comprised of two non-DS filters, each decomposable into spatial and temporal projections (profiles shown at *left* and *top*). Each non-DS filter has a rectifying nonlinearity associated with it (*right*), which is fit to the data. **B.** Performance of the two models of the simple cell from (**A**) are assessed by the log-likelihoods predicted for the left out samples in 10-fold cross-validation. The model based on separable non-DS filters (“sep”) yields comparable predictions, and thus provides a reasonable explanation of the simple cell’s DS. **C.** The two eigenvectors E1 and E2 corresponding to the two significant eigenvalues shown in Fig. **1B** (top row) are both direction selective. Furthermore, they form a “quadrature pair”, and can be described by different combinations of the two sets of spatial (*top*) and temporal (*left*) kernels via the sum of two sets of separable filters, which are obtained using singular value decomposition. As shown in Adelson & Bergen (1989) and reflected in the data, 2 spatial and 2 temporal kernels suffice to form a quadrature pair. While this model performs significantly better than the STA it clearly misses the complex selectivity captured by the model containing multiple localized features (see crossvalidation performance shown in Fig. **3B**).

Of the 17 DS simple cells in the random bar study, only two have a single excitatory filter. Such a filter has a spatiotemporal tilt indicative of DS and thus is “non-separable” (Fig. 5*A*), but can be decomposed into two non-DS filters with their spatial and temporal kernels shifted relative to each other (Fig. 5*A*, lower panel). For these simplest neurons in the recorded DS population such construction provides a model capturing the first stage of DS construction in V1 that is both in line with LGN response characteristics as well as consistent with the population data reported in this study (Fig. 4*B*). Nonlinear modeling applied to this two-filter “non-DS input” model finds each filter associated with a rectifying nonlinearity (Fig. 5*A* lower panel), and it can predict the observed response with equivalent accuracy as the one-filter DS input model (Fig. 5*B*). Although this comparison is not definitive proof that DS is constructed in this neuron, the ability of separable models to reproduce the neuron’s response as accurately as non-separable models only occurred for these neurons, and not any of the multi-filter neurons in this study.

Furthermore, the “rectifying” nonlinearities associated with each non-DS filter suggest that cortical DS can be constructed using the simple biologically plausible mechanism of integration with a spiking nonlinearity, without necessitating the more complex computations implied by abstract models of DS such as the Energy Model and the Reichardt model ([8], [40], see Discussion).

Extending this construction to the next stage of DS computation, the simplifying pair of complex cell input filters (E1 and E2, Fig. 1B) can be decomposed into four separable RFs representing different combinations of two spatial and temporal filters (see Methods, Fig. 5*C*), as predicted by the Energy Model [8].

### 2. Multiple Excitatory DS Filters Imply DS Inputs

**Figure 6:**
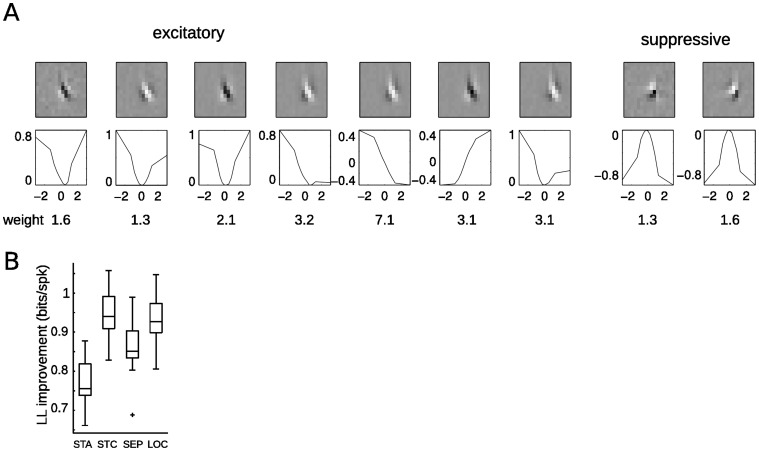
Localized features explain direction selectivity in representative simple cells. **A.** The spike triggered analysis of a typical simple cell identifies multiple excitatory and suppressive filters. The top row shows the relevant localized STRFs from the excitatory (left) and suppressive (right) subspace and the lower row the associated nonlinearities. **B.** Although clearly a simple cell based on its modulation index (MI = 1.51), comparison of cross-validated likelihood indicates that the neuron is best described by multiple filters (“STC", “loc”). Models just based on the spike triggered average (“STA”, Fig. **5A**) or models replacing the excitatory filters by non-DS inputs (as in Fig. **5A**) yield lower performance.

Most simple cells are best described using multiple excitatory and suppressive filters, illustrated here with a typical example with seven excitatory and two suppressive filters (Fig. 6*A*). The nonlinearities associated with the individual filters show a variety of shapes ranging from rectifying to bowl-shaped (note the inverted bowls in the suppressive directions). Similar to the complex cells, including these additional filters beyond the STA leads to a much better prediction of the response to random bar stimuli (Fig. 6*B*). Contrary to previous proposals based on the interplay of excitation and delayed asymmetric inhibition [41], the relative spatiotemporal placement of excitatory and suppressive filters does not account for these cells’ DS, which we could probe by shifting the relative positions of these filters (data not shown). Instead, the DS of the neuron appears to stem entirely from the spatiotemporal structure within each filter (Fig. 4*D*).

Furthermore, we found that the neuron’s response could not be as accurately predicted by models with separable inputs (Fig. 6*B*), unlike the “simplest” simple cells described above (Fig. 5*A*). This result makes sense intuitively, because a set of shifted DS filters will decompose into a set of overlapping non-DS filters without clear spatiotemporal relationships that maintain DS. While this statistical validation cannot rule out non-DS inputs, it does imply that either the neuron receives inputs that are already DS, and/or performs a more complicated computation whereby particular inputs with appropriate spatial and temporal phase relationships are separately combined nonlinearly before a second stage of nonlinear pooling across all inputs.

Thus, the presence of more than one DS excitatory filter, as well as suppressive DS filters, implies a “second-order” computation that relies on two stages of nonlinearities, with one preceding the summation of different DS inputs. The fact that most simple cells (15 out of 17) have multiple filters thus suggests that DS is constructed through multiple stages, with only a fraction of cortical neurons receiving solely non-DS input.

### 3. Pooling Local DS Subunits Explains Multiple Aspects of Phase-invariant DS in Complex Cells

**Figure 7:**
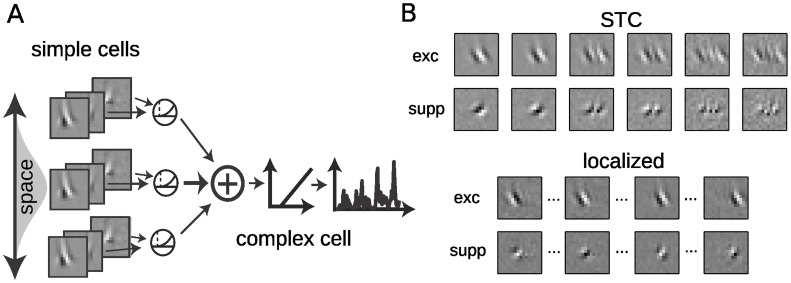
Construction of DS complex cells by pooling over inputs from typical DS simple cells. **A.** A complex cell model is constructed from a population of typical simple cell models having multiple excitatory and suppressive filters themselves. The family of simple cells is created by spatially shifting all filters of a prototypical simple cell model (see Fig. 6A) over a spatial envelope representing the complex cell receptive field. These inputs are summed and processed by a spiking nonlinearity to generate a firing rate prediction, and a Poisson spike generator then produces simulated data for further analysis. **B.** *Upper panel*: the STC filters for the simulated complex cell display a range of different spatiotemporal frequencies and opposing DS for excitatory and suppressive directions, as observed for all complex cells in this study (*e.g.*, Fig. 1E). *Lower panel*: The localized features inferred from these STC directions comprise a set of homogeneous, spatially shifted units, which closely resemble the dominant excitatory and suppressive directions from the first-order model (Fig. 6A).

The fact that a large majority of simple cells have multiple excitatory and suppressive features implies that most inputs to a complex cell likely have multiple features, which would be a more complex situation than implied by the complex cell models described earlier (Fig. 2). While we cannot infer inputs with multiple features from complex cell spike trains with this approach, we can test how the presence of multiple excitatory and suppressive filters would affect the selectivity of the resulting complex cell. We consider a “second order” model of a complex cell, constructed by pooling more typical simple cells that have multiple filters themselves: in this case the more complicated simple cell considered above (Fig. 6*A*), which has multiple excitatory and suppressive filters. As suggested by earlier analysis of complex cell inputs (Fig. 2*E*), we hypothesize that the simple-cell-like inputs onto this cell are similar in their selectivity, but spatially shifted relative to one another. Thus, our simulated complex cell is created by spatial shifts of the entire multi-filter model of the “complicated” simple cell (Fig. 7*A*), which are then additively combined, weighted by a Gaussian spatial envelope, to form a generator potential. To generate simulated spikes, the generator potential is finally passed through a spiking nonlinearity to yield a firing rate that is the input to a Poisson spike generator. The resulting spike train can be analyzed like the experimentally recorded data using the analyses presented in this work.

The extracted STC directions (Fig. 7*B*) mirror the characteristic pattern found for most complex cells: a sequence of strongly direction selective filters of increasing spatial frequency with expanding and suppressive filters being sensitive to motion in opposite directions. Note that no suppressive terms were added to the simulated neuron, suggesting that the pattern of opposing DS suppression observed in STC analyses of complex cells (Fig 1*B*; [15]) may be an emergent property resulting from the integration of the more complicated simple cell inputs, rather than direct inhibition. Further simulations indicate that the observed opponency between excitatory and suppressive filters is not constructed at this stage but derives from simple cells being suppressed by motion in the null direction (data not shown). Similar to [42] we wondered, to what degree the observed selectivity can be explained by a purely excitatory feed-forward model and our findings are consistent with the failure to observe opposite-direction inhibition onto complex cells in intracellular studies [35]. Finally, the localized filters based on these STC directions (Fig. 7*B*) match the dominant excitatory filters of the first-order inputs (Fig. 6*A*), providing further evidence that the localized solutions infer characteristic properties of the neuron’s inputs.

In summary, our results suggest that the computation performed by individual complex cells is likely constructed over several stages of cortical processing, and reflects previous nonlinear computation performed in simple cells as well as that performed by the complex cell itself.

### Estimating Receptive Fields from Responses to Naturalistic Movies

**Figure 8:**
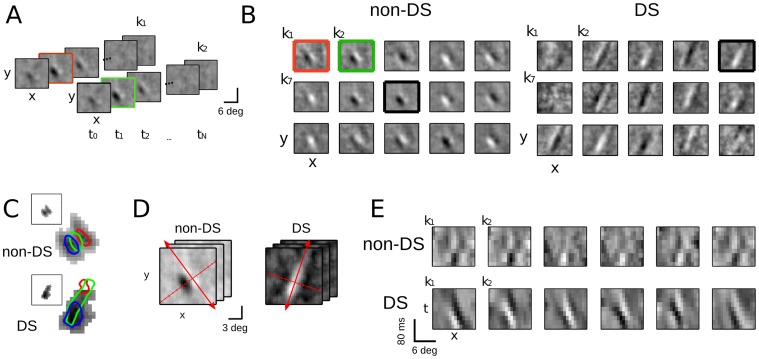
Receptive fields can be estimated from responses to naturalistic movies. **A.** Two filters found by applying the STC and localization methods to a V1 complex cell recorded in the context of naturalistic movies. To represent the three dimensions of the filters (two spatial and one temporal), the filters are shown as a series of two-dimensional (2-D) spatial plots across latencies. **B.** The set of localized filters across space for an example non-DS neuron (14,664 spikes, DSI = 0.05, *left*) and DS neuron (2,628 spikes, DSI = 0.51, *right*). Here, each filter is represented by its 2-D spatial slice at 40 ms latency, which is the latency with maximal spatial power, *i.e.*, variance across pixels (*green* and *red outlined frames* in A). The 15 spatial maps shown in each case are examples of localized filters centered on different spatial locations. **C.** A density plot showing the total temporal power across all localized filters for the non-DS (*top*) and DS (*bottom*) neurons, demonstrating that - despite using a range of locations for the localization method - localized filters only exist in a defined region. *Rectangular insets* indicate the relative size and position of the spatial filter, and the colored contours overlaid on density plot show the outline of three example localized filters. **D.** To compare with the results from the analysis on the random bars data (Figs. 1–5), spatiotemporal slices oriented in the neuron’s preferred direction (*red arrows*) are computed, and spatially projected on the axis perpendicular (*red tick marks*). **E**. Projections along the direction indicated in (D) for each neuron’s localized filters reveal *x-t* plots that are consistent with the 1-D dataset and illustrate differences in direction selectivity between the cells shown in (B) and (C).

While the one-dimensional random bar stimulus is particularly effective for identifying and characterizing the fundamental aspects of the DS computation, one might imagine that such findings might be a product of the random bar context, and not generalize to more realistic stimuli. To demonstrate that our previous results are not limited to the specific dataset, we verified that the above description holds for much more complex stimuli, using neurons recorded from cat V1 in the context of spatiotemporal pink noise and short natural movies [22]. In order to do so, we adjusted the STC analysis to account for stimulus correlations [27], [28], and then adapted the search for localized filters within the STC subspace to a two-dimensional grid of spatial positions (Fig. 8*A* and *B*) (see Methods).

Applying the adapted method to these “three-dimensional” (two dimensions of space plus one of time) naturalistic stimuli results in spatially localized filters with temporal power profiles of similar shape, which together tile a larger region of the visual field: now in both spatial directions (Fig. 8*C*). However, despite searching for localized filters over an area extending well beyond the classical receptive field, the localized filters are concentrated on a limited spatial area. To compare results of this method with earlier results derived from the random bar experiments, the preferred orientation of each neuron is estimated from the localized filters (Fig. 8*D*), and each two-dimensional spatial slice is projected along the axis perpendicular to this preferred orientation. This produces *x-t* plots (Fig. 8*E*) that are directly comparable with those measured from the random bar experiments (Figs. 1*B*, 2*B*, 4*B*).

As in the one-dimensional case, the aligned excitatory features can be succinctly described by two basis features, indicating a homogeneous population of inputs with similar properties (not shown). As with the DS neurons studied thus far, the *x*-*t* plots of the DS neuron (Fig. 8*E*, *bottom*) have a distinct spatiotemporal tilt that is similar across filters. In contrast, the filters of the non-DS cell have no tilt, and resemble those of non-DS neurons from the random bar dataset.

Thus, while the analysis of 3D receptive fields recorded in natural and naturalistic movies requires more data, the localization and modeling methods described above reveal a very similar picture to that found in the random bar data.

## Discussion

The construction of direction-selectivity (DS) in V1 by the cortical circuitry of carnivores and primates is well established by experiment, including its experience-dependent emergence during the cortical critical period [43] and the rarity of robust DS tuning observed in primate retina [44], [45]. However, unlike DS constructed in the retina of rodents [46] and in the fly [47], the construction of phase-invariant DS in cortex is a network phenomenon, involving at least two processing stages within the cortex and the likely combination of feed-forward and recurrent cortical inputs [48], [49]. Such network phenomena have led to a variety of DS models [8], [50], but provide a challenge for experimental validation, which is typically limited to recording the bulk properties of many inputs [35] or targeting single connections [51].

Our approach has been to use nonlinear modeling coupled with statistical validation to understand how the observed response properties of a given neuron to complex stimuli likely emerge from pooling over biologically plausible inputs. We focus in this study on direction-selective (DS) neurons because the construction of phase-invariant DS necessarily incorporates two types of nonlinear processing and thus more greatly constrains models that might produce it. By studying this across a population of recorded neurons ranging from simple to complex cells, our results suggest multiple nonlinear feed-forward stages of stimulus processing within V1. Our results are consistent with DS initially being constructed from non-DS inputs with particular spatiotemporal relationships (Fig. 5*A*). Similar to the construction of orientation selectivity [42], such *de novo* construction however only appears to occur in a minority of simple cells, with the rest appearing to receive at least some amount of distinct DS input, which can be both excitatory and suppressive (Fig. 6*A*). Finally, complex cells appear to be a special (although quite numerous) case, with inputs that are balanced in phase across a larger receptive field (Figs. 2*C*). Complex cell filters, and the corresponding nonlinearities, effectively extract local motion energy from different spatial locations and pool this information to get a phase-invariant estimate of motion energy over a wider portion of visual space. In fact, the apparent complexity of the STC filters observed in previous studies [15] (also Fig. 1*B*) likely reflects the scale difference between the local calculation of motion energy and the overall region of the receptive field.

These results are in line with current consensus about the construction of complex cell receptive fields [7], [52]. More specifically, they support a hierarchical model in which all but the simplest cells do not receive purely non-DS inputs, as well as the idea that simple and complex cells exist along a continuum [53]. However, our results go beyond these observations by providing a more detailed view of the properties of putative inputs, and how they are combined within the observed neuron.

Because the resulting description is based on statistical analysis of experimental data, it can only reflect which models are more consistent with the observed data. Such validation might be viewed as a complementary approach to experimentally isolating single inputs, which allows a statistical picture of a given neuron’s inputs over many paired recordings [51], [54], [55], but cannot address how they interact. However, because this modeling approach can incorporate other experimental details directly, such as the spike trains of simultaneously observed neurons (*e.g.*, [56]), it might be utilized in future experiments where multiple observed spike trains and functional modeling are combined to test these findings. In this sense, our results make a closer link between empirically measurable statistical properties of complex cell inputs and the Energy Model with its functional interpretation.

### Phenomenological versus Biological Descriptions

Phase-invariant DS clearly shows the limits of linear models to account for the DS responses of complex cells. As a result, the Energy Model [8] succinctly describes how phase-invariant DS can emerge from a simple nonlinear computation on the visual stimulus. It predicts cortical responses to a range of parametric stimuli [9], [57], and also appears to capture the basis for downstream motion processing [58], [59]. Studies using noise stimuli instead of patterned motion find quadrature pair inputs with squaring nonlinearities [14], also consistent with the basic tenets of the Energy Model.

However, previous applications of the Energy Model do not directly address how such computation is implemented by the cortical circuitry. For example, the original formulation of the Energy Model suggested combinatorial multiplicative interactions of separate spatial and temporal filtering, which might be mathematically equivalent to squaring nonlinearities applied to two quadrature pair inputs [8], [14]. While the presence of apparent quadrature pairs has been the subject of some models [60], it is likely a mathematically compact description of a much larger pool of similarly oriented filters across multiple phases and/or positions (*e.g.*, Figs. 2,7). Such a possibility was explicitly mentioned as an interpretation of the initial high-dimensional STC analysis [15] and the techniques presented here allow such a description to be derived directly from the data, consistent with other recently developed methods [61], [62]. Our results regarding DS in cat and monkey therefore contribute more detailed evidence in favor of localized energy computations previously found with other stimuli [10] but do not support alternative mechanisms such as relative placement of untuned excitation and asymmetric inhibition [41] or the multiplicative combination of filter outputs underlying the construction of DS in the original Reichardt model favored for other species [47]. Given the computational equivalence of both approaches to construct phase invariant opponent DS [8], the concrete mechanism we propose reconciles those two seemingly incompatible proposals.

### Comparisons to Alternative Nonlinear Approaches Applied to V1

Here, we model V1 responses explicitly using biologically plausible filters and nonlinearities. These constraints are introduced through the GNM model structure, which implicitly assumes summation over a neuron’s inputs, and provides straightforward methods to estimate the nonlinearities associated with individual inputs from the data. This allows for the validation of models with explicit rectification against more general nonlinearities (Fig. 5*A*), and the ability to gauge the effects of pooling recorded simple cells into complex cells (Fig. 7).

The modeling framework employed in this study is a novel hybrid of previous analyses based on spike-triggered covariance (STC) [23], [24] and maximum-likelihood methods applied to linear [31] and nonlinear [17] models. This approach can be divided into two separate components, where extensions of STC are used to find a set of localized filters using the STC subspace (Fig. 2*A*), and then the nonlinear components of the model are identified by leveraging techniques in maximum likelihood estimation [16], [17], [31] given these filters.

The first component of our method relies on STC analysis. STC filters are typically considered as identifying the important stimulus subspace that affects the neuron’s response, although the individual filters themselves do not necessarily have direct meaning in this context [23]. Previous methods have proposed searching this subspace for “directions” optimizing their informativeness [25], statistical independence [26], or maximum entropy [63]. We perform a similar search within this STC subspace but use a biologically motivated objective: the spatial localization of filters across the overall receptive field. The resulting localized estimates span the same subspace as the STC filters and possess comparable predictive performance, but provide a straightforward interpretation of the high-dimensional subspaces identified by STC [29], [61], [62]. Although our optimization objective does not explicitly constrain other RF measures (such as preferred spatial or temporal frequency), the resulting filters are also consistent with respect to these additional properties, both across the population as well as regarding other studies [18], [36], [37].

The second component of our method associates an individual nonlinearity with each filter, which can be directly estimated using maximum likelihood. While this assumption does constrain the potential computation allowable compared to high-dimensional joint nonlinearities, such as in more general formulations of STC-based models [24], these more flexible formulations are practically limited to at most joint two-dimensional nonlinearities [15], [64] due to data limitations. Furthermore, in assuming separate nonlinear functions associated with each filter, the GNM allows for the application of biologically plausible constraints, and a directly interpretable effect of each filter as the properties of potentially observable inputs onto a given V1 neuron. While this approach shares interpretability [65] and flexibility [66] with neural network approaches, the nonlinearities associated with individual subunits are estimated from the data and parameter estimation does not require specification of additional parameters like training schedule, number of hidden units, or inclusion of weight decay and momentum terms. Similar subunit based models have recently been shown to efficiently capture the nonlinearities observed at other stages of early sensory processing [62], [67], [68].

Thus, this study provides an example of how statistical approaches applied to readily available extracellular data can be used to learn about network-level computation, at a level that might be explicitly validated experimentally. Explaining the construction of DS responses in different cells ranging from simple to complex, our results more generally provide an important step to understand how increasingly abstract information is extracted across different cortical areas to form invariant representations. Determining the inputs to a neuron and modeling how they are combined forms an important part of understanding how specific computations are implemented by the interplay of individual cells in the circuits they are embedded in.
